# Hepatitis E Virus Genotype 3 Diversity, France

**DOI:** 10.3201/eid1501.080296

**Published:** 2009-01

**Authors:** Florence Legrand-Abravanel, Jean-Michel Mansuy, Martine Dubois, Nassim Kamar, Jean-Marie Peron, Lionel Rostaing, Jacques Izopet

**Affiliations:** Institut National de la Santé et de la Recherche Médicale, Toulouse, France (F. Legrand-Abravanel, M. Dubois, L. Rostaing, J. Izopet); Centre Hospitalier Universitaire, Toulouse (F. Legrand-Abravanel, J.-M. Mansuy, M. Dubois, N. Kamar, J.-M. Peron, L. Rostaing, J. Izopet)

**Keywords:** Hepatitis E virus, genotype 3, full-length genome, genetic diversity, France, dispatch

## Abstract

We characterized 42 hepatitis E virus (HEV) genotype 3 strains from infected patients in France in 3 parts of the genome and sequenced the full-length HEV genotype 3f genome found in Europe. These strains are closely related to swine strains in Europe, which suggests zoonotic transmission of HEV in France.

Hepatitis E is a water-borne infection in developing countries and is believed to spread zoonotically in industrialized countries (*1*). Hepatitis E virus (HEV) is a positive-sense RNA virus that belongs to the family *Hepeviridae* (*2*). The coding region consists of 3 discontinuous open reading frames (ORFs). One region within ORF1, the hypervariable region, displays substantial genetic diversity (*2*). HEV strains are classified into 4 genotypes, and it was recently proposed that HEV genotypes are divided into 24 subtypes (*3*). Although genotypes 1 and 2 have been found exclusively in humans, genotypes 3 and 4 have been found in humans and animals such as pigs, boars, and deer. Genotypes 1 and 2 have been isolated in tropical and subtropical countries in Asia, Africa, and America; genotype 4 has been found only in Asia. Genotype 3 has been identified almost worldwide, but the distribution of its 10 subtypes varies greatly. Subtypes 3a and 3j strains have been mainly identified in North America; 3b, 3d, and 3g strains in Asia; and 3c, 3e, 3f, 3h, and 3i strains in Europe (*3*). The finding of several cases of HEV infection in southern France (*4*,*5*) prompted us to characterize the diversity of the strains and to determine the full-length sequence of the most prevalent genotype in this area.

## The Study

We studied 42 HEV strains from patients at Toulouse University Hospital, France. None of the patients had traveled abroad during the previous 6 months or reported any contact with pigs before the onset of the disease. HEV infection was determined by detecting HEV RNA by using molecular tools (*5*). We sequenced 2 fragments within ORF1, the hypervariable region, and the RNA-dependent RNA polymerase (RdRp) genes as previously described (*6*,*7*). A 348-nt fragment of ORF2 was amplified according to the protocol of Inoue et al. (*8*). The whole genome was amplified with 6 overlapping reverse transcription–PCRs. The primers are listed in Table 1. The reverse transcription conditions were 50°C for 30 min and 85°C for 5 min. The PCR cycling conditions were initial denaturation at 94°C for 2 min, then 50 cycles of denaturation at 94°C for 15 sec, annealing at 60°C for 30 sec, and elongation at 68°C for 3 min.

**Table 1:** Primers used to amplify the whole hepatitis E virus genotype 3f genome

Fragment size, bp	Nucleotide position*	Sense primers (5′ → 3′)	Antisense primers (5′ → 3′)
990	1–990	TAGGCAGACCACGTATGTGGTCGATGCCATGGA	gccggtcccagatRtgSaccggRa
1,294	878–2172	ACAGAGGTGTATGTTAGATCCATATTTGGC	GGGGAGAAGTCGCTAGAGAAACCTGATGT
2568	2001–4569	CCCAGCGSCWTTCGCTGACCGG	CGGATAAGCCACTGGGGCATGCCRCACT
736	4542–5278	AGTGYGGCATGCCCCAGTGGCTTATCCG	GCCGGTGGCGCGGGCAGCATAGGCA
1,480	5003–6484	ACGAATGTYGCGCAGGTYTGTGT	cccttrtcctgctgngcattctcgacaga
964	6363–7327	GACAGAATTRATTTCGTCGGC	TTTCCMGGGRGCGCGGAACCCCGAA

*Nucleotide position refers to Burma strain, M73218.

**Figure 1:**
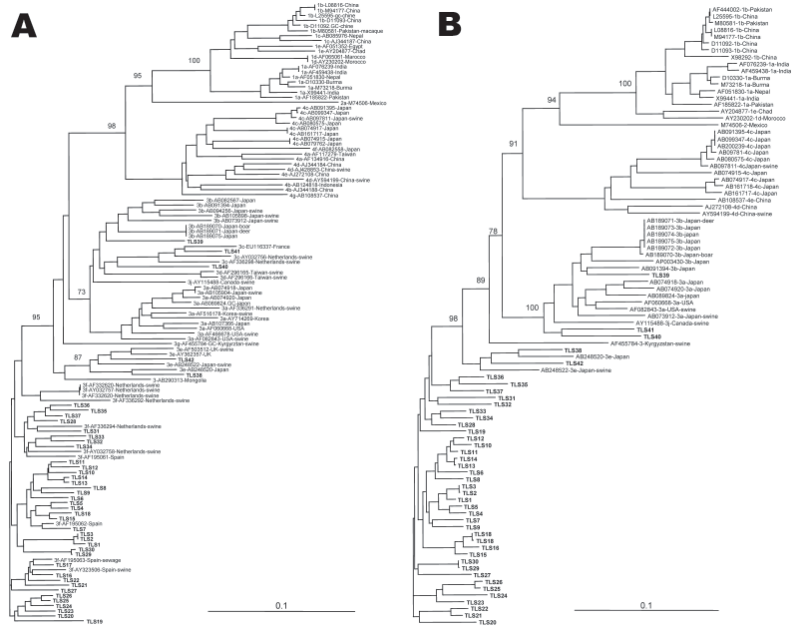
Phylogenetic relationship among hepatitis E virus (HEV) strains from southwestern France and reference strains available in GenBank based on a 348-nt sequence in the open reading frame 2 (A) and on a 383-nt sequence of HEV RNA–dependent RNA polymerase (B). Genetic distances were calculated by using the Kimura 2-parameter method; phylogenetic trees were plotted by the neighbor-joining method. The reproducibility of the branching pattern was tested by bootstrap analysis (1,000 replicates). Each branch was labeled with the GenBank accession number of the strain, the geographic area, and the host, if nonhuman, and the geographic area where the sequence was isolated. The genotype and subtype were identified according to Lu et al. (*3*). Scale bars represent nucleotide substitutions per site. **Boldface** indicates the G3 French strains.

The phylogenetic tree obtained for the ORF2 region showed that all strains belonged to genotype 3 (Figure 1, panel A). A total of 37 strains (TLS1–TLS37) segregated as a distinct clade among genotype-3 reference sequences, whereas 5 other strains (TLS38–TLS42) formed distinct branches in the tree. TLS1–TLS37 could be subtyped as 3f, according to the classification proposed by Lu et al. (*3*). The nucleotide sequences of our local 3f strains and the strains from Spain and the Netherlands, which had been identified in humans, pigs, and sewage of animal origin, had 90.2%–95.3% identity. The 5 remaining strains were also genetically related to swine strains. Strains TLS38 and TLS42 were more closely related to 3e strains from swine in Great Britain (88.5%–92.1% nucleotide sequence identity). These strains were also closely related to Japanese strains AB248520 and AB248522, which may have a British origin (87.5%–93.4% identity) (*9*). Strains TLS40 and TLS41 were located on the same branch as swine HEV isolates identified in the Netherlands and classified as subtype 3c. The nucleotide sequence of TLS41 had 91.7%–92.7% identity with that of strains from pigs in the Netherlands, whereas that of TLS40 was only 85.5%–86.1% identical to that of pig strains from the Netherlands. Strain TLS39 was similar to the 3b strains identified in Japan in human patients or animals (86.8%–90.1% nucleotide sequence identity).

The topology of the phylogenetic tree obtained for the RdRp region was similar to that of ORF2 (Figure 1, panel B). All the strains clustered with genotype 3 strains. The same 37 strains (TLS1–TLS37) clustered together (87.9%–99.1% nucleotide sequence identity) and the same 5 strains (TLS38–TLS42) appeared to be more divergent.

**Figure 2:**
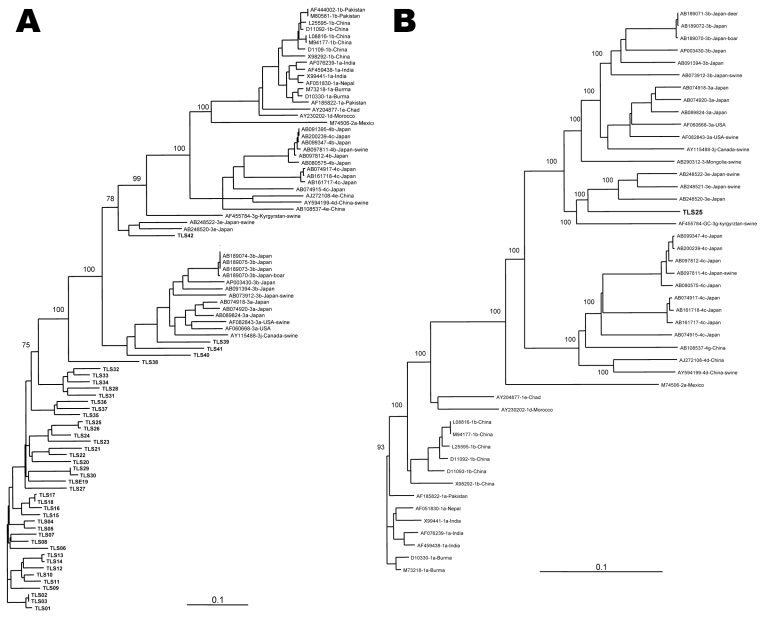
Phylogenetic relationship among hepatitis E virus (HEV) strains from southwestern France based on a 345-nt sequence of HEV hypervariable region (A) and on the full-length sequence of TLS25 and HEV strains whose entire sequence is known (B). Genetic distances were calculated by using the Kimura 2-parameter method; phylogenetic trees were plotted by the neighbor-joining method. The reproducibility of the branching pattern was tested by bootstrap analysis (1,000 replicates). Each branch was labeled with the GenBank accession number of the strain, the geographic area, and the host, if nonhuman, and the geographic area where the sequence was isolated. The genotype and subtype were identified according to Lu et al. (*3*). Scale bars represent nucleotide substitutions per site. **Boldface** indicates the G3 French strains.

The hypervariable region gave the same clade of 37 strains in the phylogenetic tree, whereas the 5 remaining strains were more divergent (Figure 2, panel A). Strains TLS1–TLS37 exhibited only 70.1%–86.8% nucleotide sequence identity in this part of the genome, which shows the great diversity of this region. According to the primers position (*6*), a fragment of 345 nt was expected for all the strains. Actually, the fragments obtained after amplification of the hypervariable region of the genotype 3f strains varied from 435 nt for 29 samples, to 412 nt for 3 samples, and to 348 nt for 5 samples. For the 3e strains, the fragments were 387 nt and 412 nt. The PCR product from the hypervariable region was 345 nt for only the 3c and 3b strains.

The complete genome from TLS25 strain (genotype 3f) was amplified. The genomic length was 7,321 nt with the 3 major ORFs. Comparison with full-length reference sequences showed an insertion of ≈90 nt in the hypervariable region. Phylogenetic analysis of the sequences of HEV genotypes 1–4 strains confirmed that TLS25 belonged to genotype 3 and was genetically distinct from the genotype 3 strains found in Asia and in the United States (Figure 2, panel B). Comparisons with the complete genome sequences of HEV indicated that strain TLS25 shared 72.0%–72.9% nucleotide sequence identity with genotype 1 strains, 72% nucleotide sequence identity with genotype 2 strains, and 73.7%–74.5% nucleotide sequence identity with genotype 4 strains (Table 2). Strain TLS25 shows 83.1%–84.3% nucleotide sequence identity with HEV genotype 3e, 81.8% identity with the HEV strain 3g, 79.7%–80.3% identity with HEV genotype 3b, and 79.5%–81.8% identity with HEV genotype 3a over the entire genome. No recombination event was detected within the TLS25 strain by using the Recombinant Detection Program (http://darwin.uvigo.es/rdp/rdp.html). The sequences were deposited in GenBank under accession nos. EU495106–EU495232.

**Table 2:** Nucleotide sequence identity between TLS25 (hepatitis virus E genotype 3f) and available full-length genomes

GenBank accession no.	Country	Origin	Genotype	Sequence homology, %
M73218	Burma	Human	1a	72.7
D10330	Burma	Human	1a	72.8
AF051830	Nepal	Human	1a	72.7
AF076239	India	Human	1a	72.3
X99441	India	Human	1a	72.6
AF459438	India	Human	1a	72.5
AF185822	Pakistan	Human	1a	72.3
L08816	China	Human	1b	72.5
M94177	China	Human	1b	72.7
L25595	China	Human	1b	72.7
D11093	China	Human	1b	72.5
D11092	China	Human	1b	72.8
X98292	China	Human	1b	73.0
AY230202	Morocco	Human	1d	72.9
AY204877	Chad	Human	1e	72.1
M74506	Mexico	Human	2a	72.0
AB200239	Japan	Human	4c	74.2
AB099347	Japan	Human	4c	74.2
AB097811	Japan	Swine	4c	74.3
AB097812	Japan	Human	4c	74.2
AB080575	Japan	Human	4c	73.6
AB074915	Japan	Human	4c	74.5
AB074917	Japan	Human	4c	74.4
AB161717	Japan	Human	4c	74.1
AB161718	Japan	Human	4c	74.1
AB108537	China	Human	4g	74.4
AJ272108	China	Human	4e	73.7
AY594199	China	Swine	4d	74.1
AF455784	Kirgizstan	Swine	3g	81.8
AB074918	Japan	Human	3a	80.4
AB074920	Japan	Human	3a	80.1
AB089824	Japan	Human	3a	80.6
AF082843	USA	Swine	3a	80.5
AF060668	USA	Human	3a	79.5
AB189070	Japan	Boar	3b	80.1
AB189071	Japan	Deer	3b	79.9
AB189072	Japan	human	3b	79.7
AP003430	Japan	human	3b	80.1
AB073912	Japan	Swine	3b	80.2
AB091394	Japan	Human	3b	80.3
AY115488	Canada	Swine	3j	80.2
AB248522	Japan	Swine	3e	84.2
AB248521	Japan	Swine	3e	83.1
AB248520	Japan	Human	3e	84.3
AB290312	Mongolia	Swine	3	79.7

## Conclusions

Most (88%) of the HEV strains in France belonged to 3f subtype, but 3c, 3e, and 3b strains were also identified. Phylogenetic analyses indicated that HEV strains in France were related to swine strains previously identified in Europe. This full-length sequencing of an HEV genotype 3 strain from Europe showed it to be distinct from the genotype 3 strains found on other continents, which illustrates the great diversity of this genotype. Due to an insertion in the hypervariable region, the TLS25 sequence is longer than other HEV sequences available in GenBank. A variation in the length of the hypervariable region in 1 human strain of genotype 3e was previously reported (*9*). Because the function of this region of the genome is still unknown, the effect of such insertions on virus biology has yet to be elucidated. Zhai et al. reported that phylogenetic analyses within the RdRp region correlated well with the results from the phylogenetic analyses of the complete genome (*7*), whereas Lu et al. found that the ORF2 region was the region that determined most accurately genotypes and subtypes (*3*). Our data indicate that both regions can be used to determine the genotype and subtype. The source of autochthonous hepatitis E infection in industrialized countries is unknown. One hypothesis, supported by molecular epidemiologic studies, is that it is an emerging zoonotic infection (*10*,*11*). Contamination with HEV may be linked to occupational exposure (*12*,*13*), consumption of undercooked meat (*14*,*15*), or exposure to a contaminated environment.

Our study characterized several human HEV strains and sequenced the full-length HEV 3f genome. These strains were closely related to European swine strains. Prospective in-depth epidemiologic studies based on structured interviews are ongoing to clarify the routes of transmission in southwest France.

## References

[1] Emerson SU, Purcell RH. Hepatitis E virus. Rev Med Virol. 2003;13:145–54. 10.1002/rmv.384 12740830

[2] Okamoto H. Genetic variability and evolution of hepatitis E virus. Virus Res. 2007;127:216–28. 10.1016/j.virusres.2007.02.002 17363102

[3] Lu L, Li C, Hagedorn CH. Phylogenetic analysis of global hepatitis E virus sequences: genetic diversity, subtypes and zoonosis. Rev Med Virol. 2006;16:5–36. 10.1002/rmv.482 16175650

[4] Kamar N, Selves J, Mansuy JM, Ouezzani L, Peron JM, Guitard J, Hepatitis E virus and chronic hepatitis in organ-transplant recipients. N Engl J Med. 2008;358:811–7. 10.1056/NEJMoa0706992 18287603

[5] Mansuy JM, Peron JM, Abravanel F, Poirson H, Dubois M, Miedouge M, Hepatitis E in the south west of France in individuals who have never visited an endemic area. J Med Virol. 2004;74:419–24. 10.1002/jmv.20206 15368508

[6] Kabrane-Lazizi Y, Zhang M, Purcell RH, Miller KD, Davey RT, Emerson SU. Acute hepatitis caused by a novel strain of hepatitis E virus most closely related to United States strains. J Gen Virol. 2001;82:1687–93. 11413380 10.1099/0022-1317-82-7-1687

[7] Zhai L, Dai X, Meng J. Hepatitis E virus genotyping based on full-length genome and partial genomic regions. Virus Res. 2006;120:57–69. 10.1016/j.virusres.2006.01.013 16472882

[8] Inoue J, Takahashi M, Yazaki Y, Tsuda F, Okamoto H. Development and validation of an improved RT-PCR assay with nested universal primers for detection of hepatitis E virus strains with significant sequence divergence. J Virol Methods. 2006;137:325–33. 10.1016/j.jviromet.2006.07.004 16901555

[9] Inoue J, Takahashi M, Ito K, Shimosegawa T, Okamoto H. Analysis of human and swine hepatitis E virus (HEV) isolates of genotype 3 in Japan that are only 81–83% similar to reported HEV isolates of the same genotype over the entire genome. J Gen Virol. 2006;87:2363–9. 10.1099/vir.0.81912-0 16847132

[10] Meng XJ, Purcell RH, Halbur PG, Lehman JR, Webb DM, Tsareva TS, A novel virus in swine is closely related to the human hepatitis E virus. Proc Natl Acad Sci U S A. 1997;94:9860–5. 10.1073/pnas.94.18.9860 9275216 PMC23282

[11] Banks M, Bendall R, Grierson S, Heath G, Mitchell J, Dalton H. Human and porcine hepatitis E virus strains, United Kingdom. Emerg Infect Dis. 2004;10:953–5. 15200841 10.3201/eid1005.030908 PMC3323225

[12] Colson P, Kaba M, Bernit E, Motte A, Tamalet C. Hepatitis E associated with surgical training on pigs. Lancet. 2007;370:935. 10.1016/S0140-6736(07)61441-X 17869631

[13] Drobeniuc J, Favorov MO, Shapiro CN, Bell BP, Mast EE, Dadu A, Hepatitis E virus antibody prevalence among persons who work with swine. J Infect Dis. 2001;184:1594–7. 10.1086/324566 11740735

[14] Li TC, Chijiwa K, Sera N, Ishibashi T, Etoh Y, Shinohara Y, Hepatitis E virus transmission from wild boar meat. Emerg Infect Dis. 2005;11:1958–60. 16485490 10.3201/eid1112.051041 PMC3367655

[15] Tei S, Kitajima N, Takahashi K, Mishiro S. Zoonotic transmission of hepatitis E virus from deer to human beings. Lancet. 2003;362:371–3. 10.1016/S0140-6736(03)14025-1 12907011

